# Past, present and future of the two-spotted stink bug (*Perillus bioculatus*) in Europe revealed by citizen science

**DOI:** 10.1038/s41598-024-72501-0

**Published:** 2024-09-14

**Authors:** Péter Kóbor, Daniel Brhane

**Affiliations:** 1 Department of Zoology, HUN-REN Centre for Agricultural Research Plant Protection Institute, Budapest, 1022 Hungary; 2 Department of Integrated Plant Protection, Hungarian University of Agriculture and Life Sciences (MATE) Institute of Plant Protection, Gödöllő, 2100 Hungary; 3 Department of Plant Protection, Hamelmalo Agricultural College, P.O. Box: 397, Keren, Eritrea

**Keywords:** Heteroptera, Invasive species, Pest biocontrol, Citizen science, Ecological niche modelling, Agroecology, Invasive species, Entomology

## Abstract

The introduction of the Nearctic predaceous stink bug species, (*Perillus bioculatus*) was attempted multiple times in various countries throughout Europe to mitigate the damage caused by the invasive and harmful pest species, the Colorado potato beetle (*Leptinotarsa decemlineata*). Though these attempts were thought to be unsuccessful for decades, more recent data elucidated that the species have established small self-sustaining populations in the Balkans Peninsula, Southern Russia, and Türkiye and recently began to expand. In the past years, the European range of the species reached Eastern Europe. After the first individuals were found in Hungary in October 2023 a citizen science campaign was launched to investigate the distribution of the species in the country. By June 2024 it became evident that the species is established throughout the country. Furthermore, observations regarding beetle larvae and moth caterpillars as alternative prey were reported supporting the previous assumptions that the naturalization and expansion of the species in Europe is facilitated by dietary drift. Here, we summarize the knowledge on the European presence of the two-spotted stink bug and formulate hypotheses regarding its future distribution and the impact of the species on the insect communities of the newly colonized areas.

## Introduction

**Fig. 1:**
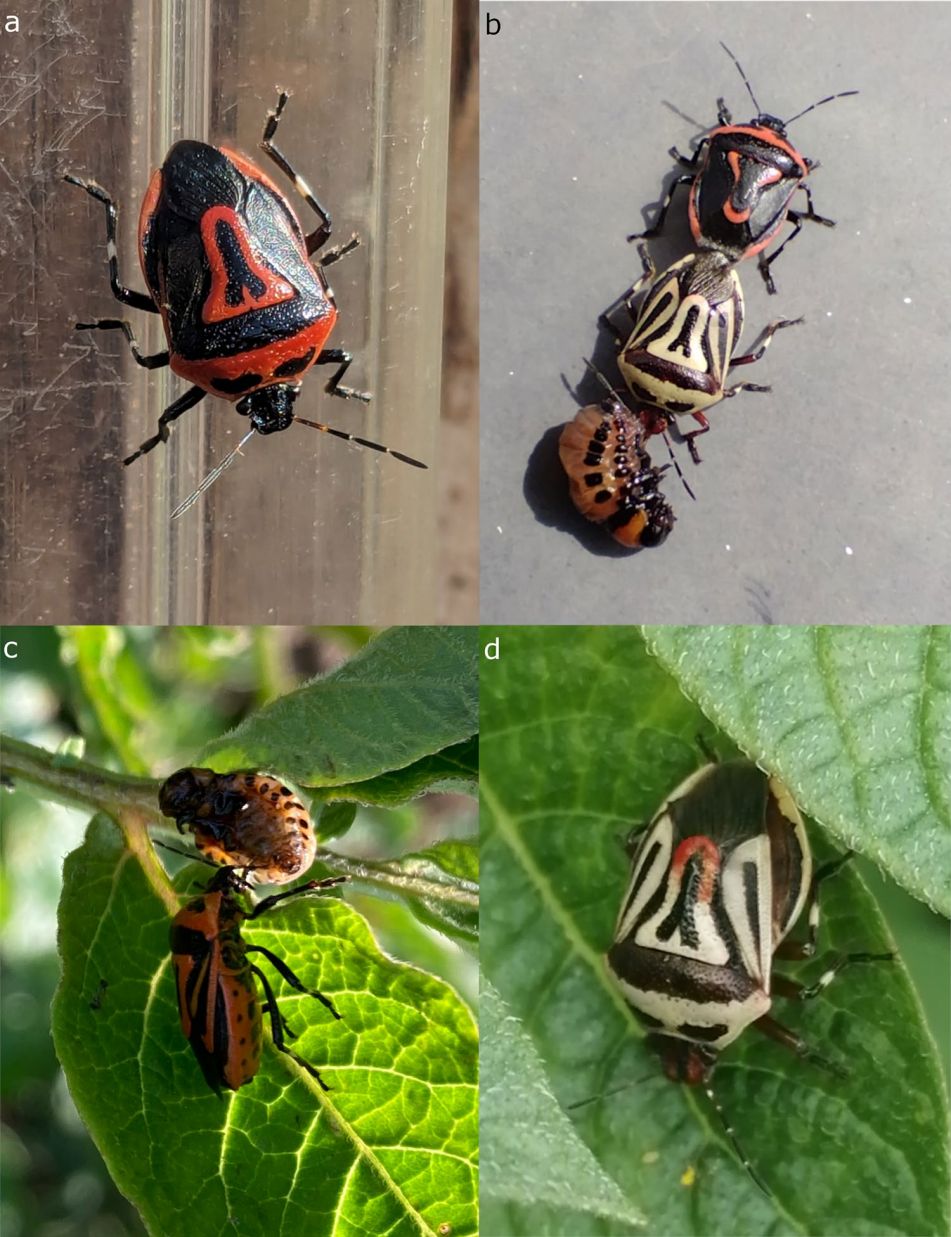
Citizen science observations of the two-spotted stink bug in Hungary: (**a**). red colour morph (associated record: PERBIO_HUN_010), (**b**). instance of simultaneous mating and predation (associated record: PERBIO_HUN_030), (**c**). instance of predation (associated record: PERBIO_HUN_020), (**d**). pale colour morph (associated record: PERBIO_HUN_029) (Identifiers refer to the records included in Supplementary Data S1).

The two-spotted stink bug [*Perillus bioculatus* (Fabricius, 1775)] (Fig. 1a–d), a North American predatory stink bug species (Heteroptera: Pentatomidae: Asopinae), is an effective and extensively studied biocontrol agent of the Colorado potato beetle [*Leptinotarsa decemlineata* (Say, 1824)], an invasive leaf beetle species (Coleoptera: Chrysomelidae) causing serious damage in potato culture throughout Europe^1–3^. The effectiveness of the two-spotted stink bug can be attributed to the following life history characteristics: the species can be multivoltine under favourable climatic conditions^4^, and both nymphal and adult stages voraciously feed on larvae and adults of the Colorado potato beetle^1,5^, the two-spotted stink bug is attracted to the volatiles released by the damaged potato plant that facilitates location of prey^6^. Furthermore, field experiments concluded that a relative abundance of 5–15 individuals/ m^2^ is sufficient to eliminate the need for the use of insecticides to control the populations of Colorado potato beetle^5,7–9^. These characteristics led to efforts to introduce the two-spotted stink bug to Europe to mitigate the yield loss caused by the defoliation resulting from the feeding of the potato beetle^9^. The first attempts to release captively bred populations took place in France in the 1930s^10^ but were disrupted by World War II. Subsequently, further experiments were performed during the 1960s and 1970s in various European countries, e.g., Czechoslovakia, France (repeated), Hungary, Italy, Poland, the USSR, and Yugoslavia^4,5, 9, 11–16^. The naturalization of the species was considered rather unsuccessful, and the failure was attributed to the disadvantageous climatic^9^ conditions and the asynchrony in the spring emergence of predator and prey^15,16^, i.e., emerging adults of the two-spotted stink bug were unable to survive in the absence of prey. The introduction attempts were discontinued, and the two-spotted stink bug was thought to be vanished from Europe. However, later suggestions were made on the possible survival and establishment of small, inconspicuous populations of the insect in the Balkans Peninsula, the southern part of Russia, Ukraine and Türkiye^2,15, 17, 18^. Until the present day, the presence of the two-spotted stink bug was reported in the following European countries: Bulgaria^19^, Greece^2^, Romania^20^, Moldavia^21^, the Russian Federation^15^, Serbia^22,23^ and Türkiye^18,24^. The survival, naturalization and range expansion of the species are hypothesized to be associated with both climate change and the dietary drift observed^15,16, 21, 23^. Latter means the utilization of alternative prey species as recorded first in the case of the ragweed leaf beetle, *Zygogramma suturalis* (Fabricius, 1775) and the olive-shaded bird-dropping moth, *Tarachidia candefacta* (Hübner, 1813), both species are feeding on common ragweed (*Ambrosia artemisifolia*) and were introduced to the southern parts of Russia to control the populations of this invasive weed species^15^. Later predation of the species was observed on leaf beetle species *Chrysomela populi* Linnaeus, 1758 and *Chrysolina herbacea* (Dufschmid, 1825)^24,25^, both species are native to Europe. Most recently the two-spotted stink bug was reported to be feeding on the larvae of the ragweed specialist leaf beetle species, *Ophraella communa* LeSage, 1986^23^. As the two-spotted stink is of very characteristic and conspicuous appearance, i.e., can be identified securely on sight, the increasing activity of citizen scientists resulted in numerous records for the species recently submitted on platforms like iNaturalist.org^26^ and on the Facebook social media platform. The latter revealed the presence of the species in Hungary in October 2023. As the invaluable contributions of citizen scientists were proven by the cases of multiple invasive species of characteristic appearance [e.g., 27–29], we launched a campaign in March 2024 to investigate the status of the species in Hungary. We combined the resulting data with records submitted to citizen science platforms and the available literature and analysed with the implication of geographic information systems and ecological niche modelling. Here, we present the results of this study that offered elaborate knowledge to assess the current status and formulate hypotheses regarding the future of the two-spotted stink bug in Europe.

## Results

### Citizen science data

**Fig. 2:**
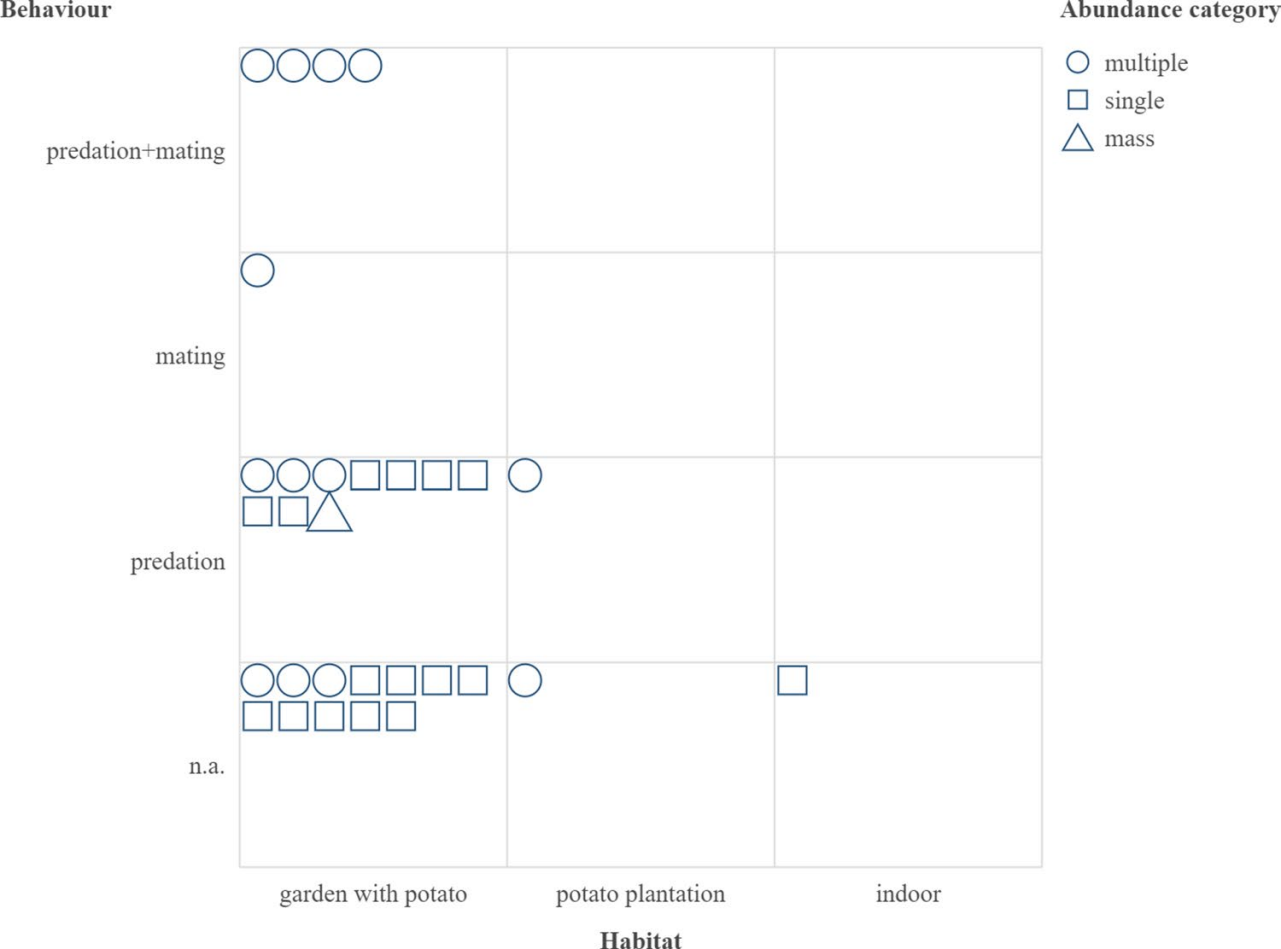
Summary of citizen science observations on abundance, behaviour and habitat.

Out of the 30 submitted observations (details can be found in Supplementary Data S1) 27 included photographs of the observed insects. 14 included the observation of a single individual, 15 of them reported the simultaneous presence of multiple individuals and one observation reported a mass occurrence of more than 20 individuals (Fig. 2). 7 of the observations of numerous individuals did not specify the observed number but phrases ‘more’ or ‘several’ were included in the report, thus we decided to include them in this category. Most observations were made in gardens with potatoes planted (17 observations), one individual was observed inside a house in January and two observations of multiple individuals was reported from a commercial potato plantations. The single occurrence that was reported from a residential building that is accidental rather than the phenomenon of hibernation in buildings. In terms of observation of specific behaviour in 14 records no indication of such was included, i.e., the observed insect was crawling on the substrate or was stationary. In 11 cases predation was observed (Fig. 1b–c) including an observation of feeding on the caterpillar of the turnip moth, *Agrotis segetum* (Dennis & Schiffmüller, 1775) (Lepidoptera: Noctuidae). One observation reported mating; in four cases, both mating and feeding were reported (Fig. 1b).

The mass occurrence was reported from the garden, and both observations in the potato plantation reported the presence of multiple individuals. Observations of specific behaviours were predominantly reported in gardens (15 observations) except a record of predation observed in potato plantations. The report on mass occurrence included the observation of predation. Instances with both mating and predation observed were reported from gardens (Fig. 2).

### Distribution history of *P. bioculatus* in Europe

**Fig. 3:**
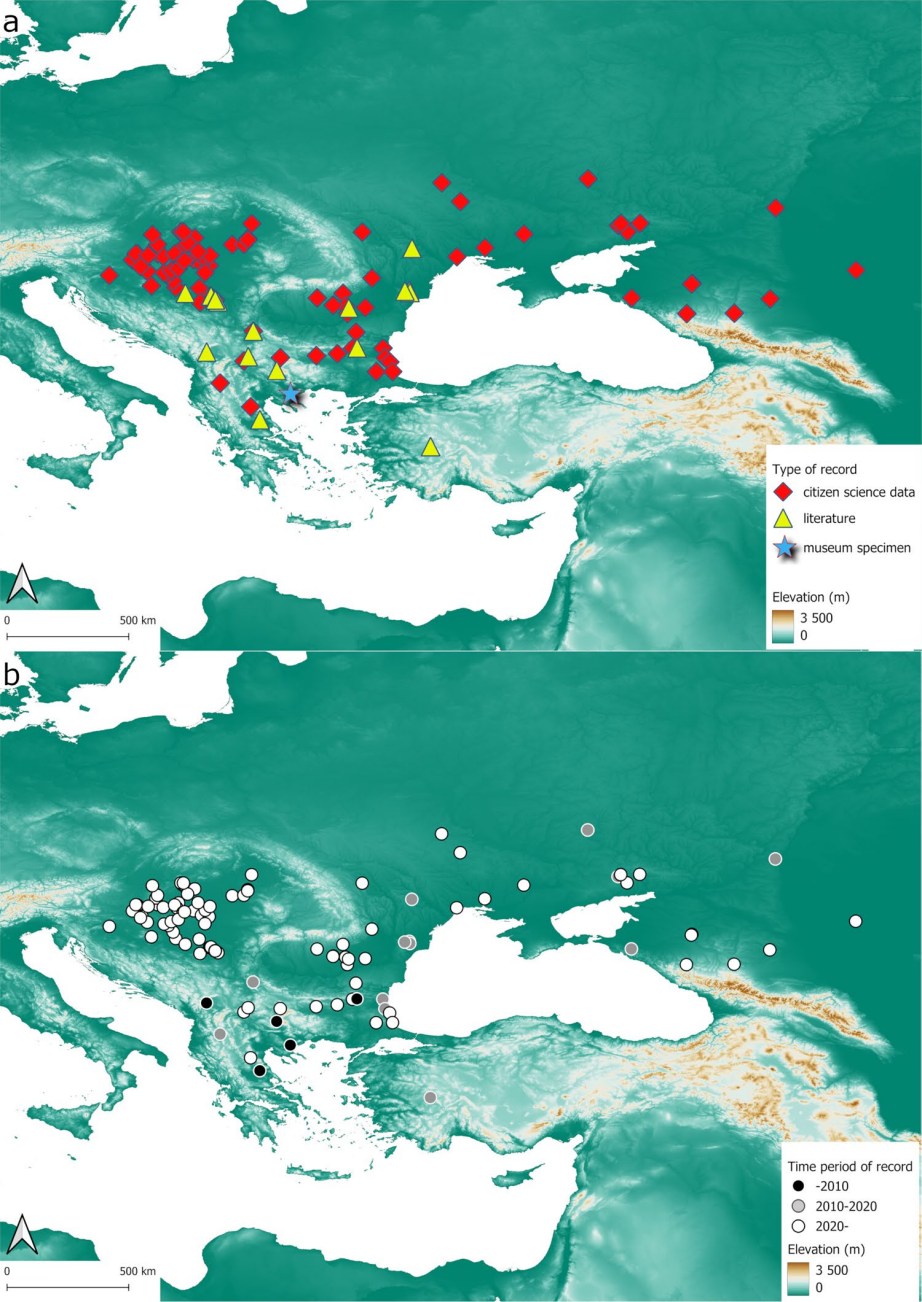
Distribution maps of the two-spotted stink bug in Europe: (**a**). records categorized by data source, (**b**). historical overview (maps were generated using QGIS 3.34.7 “Prizren” software).

The 145 individual records compiled (Supplementary Table S2) consisted of one specimen deposited in the Hemiptera collection of HNHM, 27 previously published records from literature, and 117 citizen science records (80,7%) (Fig. 3a). From the latter 87 were acquired from citizen science platforms and 30 resulted by the campaign launched by the authors. The five occurrences recorded before 2010 originated from the museum specimen and literature data. Seventeen occurrences were recorded between 2010 and 2020 of which 11 were acquired from the citizen science database iNaturalist.org (64. 7%) and 6 were published in articles regarding the European presence of the species. After 2020, 17 records were found in the literature and 106 occurrences were provided by citizen science contributors (86.2% of the 123 post-2020 records).

The compiled distribution data shows that *P*. *biolculatus* is established in Bulgaria, Greece, Hungary, Moldavia, Russia, Serbia, Türkiye, and Ukraine. From Hungary, the presence of the species was not indicated until October 2023 when observations of an unknown true bug were published in an insect identification-themed Facebook group. The submitted records show that the species is established and widely distributed in the country. The pinned specimen found in the collection of HNHM represents the earliest known specimen of the species collected in in Europe – presumably in a natural or semi-natural habitat – after the repeated and rather unsuccessful attempts of introduction (Fig. 3a). The pre-2010 records originate from Bulgaria, Greece and Serbia corroborating the hypothesis that species have survived in small populations throughout the Balkans Peninsula and Türkiye (Fig. 3b). Between 2010 and 2020 the species were recorded additionally in Moldavia, Southwest Russia and in Ukraine. The post-2020 records suggest both the local increase of abundance in the previously colonized regions and an expansion in the north-northwestern direction (Fig. 3b). When interpreting the data using the Koeppen–Geiger climatic classification dataset, the records are originating predominantly from arid cold steppes (BSk), temperate (Cfa) and cold (Dfa) zones with no dry season and hot summer (Supplementary Fig. S3) that corresponds the conditions found in the native area of the species.

### Results of the ecological niche modelling

**Fig. 4:**
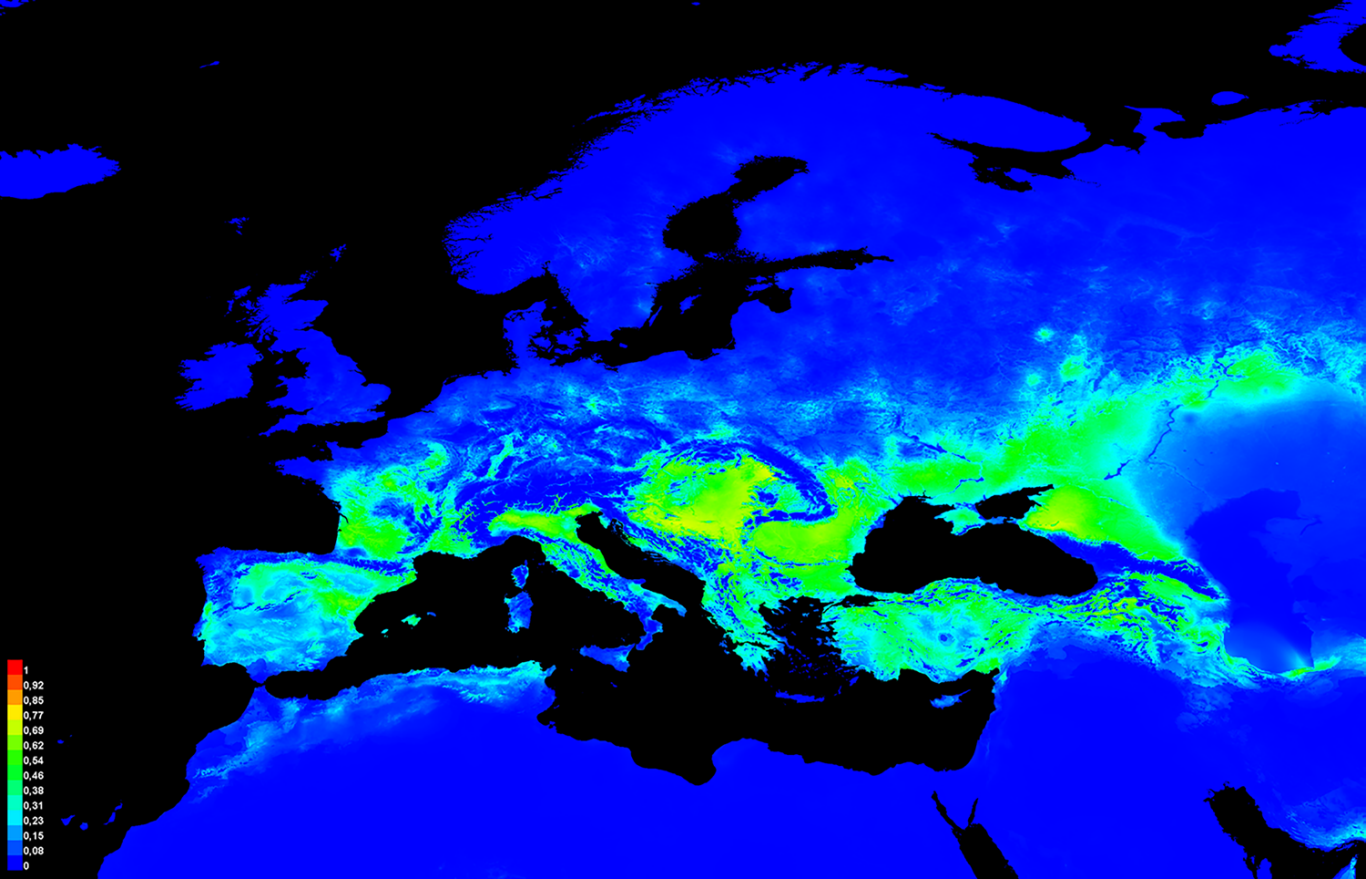
Habitat suitability in Europe resulted from the average of 10 equally robust ENM models (values ranging between 0–1 where 0 is completely unsuitable and 1 is 100% suitable) (map was generated using Maxent software, version 3.4.3).

The area under the curve in the threshold-independent receiver operation characteristic (AUC) evaluation showed a very good performance of the constructed models with an average value of 0.916 (standard deviation: 0.004). The variables with highest relative contribution to the model were the maximum temperature of the warmest month (bio5: 41.0%), the minimum temperature of the coldest month (bio6: 19.4%), and the precipitation of the driest month (bio14: 17.5%) and the Jackknife analysis of predictor importance revealed that the variable ‘maximum temperature of the warmest month’ (bio5) provides the most useful information when used in isolation and the precipitation of the driest month (bio14) when omitted. The variables ‘annual mean temperature’ (bio1) and ‘minimum temperature of the coldest month’ (bio6) were found to be the second and third most important predictors in both cases (Supplementary Fig. S4). The response curves revealed that there is a positive correlation between the annual mean temperature (bio1) and the occurrence of *P*. *bioculatus*, contrastingly, a negative response is shown for low and high values of the temperatures of the warmest and coldest months (bio5, bio6) (Supplementary Fig. S4). The models showed that from the regions of Europe currently not inhabited by *P. bioculatus* the most suitable (probability of habitat suitability ≥ 0.5) are the foothill areas of the Carpathians and the Eastern Alps (Burgenland and Lower Austria federal states of Austria; South Bohemian region of the Czech Republic; lower parts of Bratislava, Trnava, Trenčín, Nitra, Banská Bystrica and Košice regions of Slovakia; Zakarpatska region of Ukraine) the northern, pre-alpine part of Italy (mostly Friuli-Venezia Giulia, Veneto, Trentino-Alto Adige, Emili-Romagna, Lombardy, and Piedmont regions), southern and middle parts of France (parts of Nouvelle-Aquitaine and Occitanie regions) and the northeastern parts of the Iberian Peninsula (parts of Catalonia, Aragon, and Castille and Leon regions) (Fig. 4). Moderately and less suitable areas (probability of habitat suitability < 0.5) are found in Central Italy, north of France, Southern Germany, and Poland. The projection of the Koeppen–Geiger climatic classification maps implies a cut-off value of 0.5 for suitability to explore the most suitable predicted area corresponding to the projections done for the existing records in terms of climatic zones with the addition of temperate regions with no dry season and warm summer (Cfb) (Supplementary Fig. S3).

## Discussion

A citizen science campaign was launched after the first reported occurrences of the Nearctic two-spotted stink bug, *Perillus bioculatus* in Hungary (October 2023). More than half of the 30 submitted records indicated the presence of multiple individuals at the site of observation and on one occasion mass occurrence (> 20 individuals) was reported (Supplementary Data S1), thus it is to be concluded that species is established and widely distributed in Hungary. Combining the knowledge on the distribution of the species derived from museum specimens, literature data, citizen science platforms and data of the citizen science campaign revealed that *P*. *bioculatus* is widely established in the southeastern parts of Europe and is suspected to be expanding in the north-northwestern direction (Fig. 3b). The environmental niche models built from these data suggest that the distribution of *P*. *bioculatus* is closely associated with three bioclimatic variables, the temperature of the warmest, minimum temperature of the coldest months, and the precipitation of the driest month. Response curves revealed that there is a positive correlation between higher mean temperatures and the suitability of the habitats, however, high values of maximum temperatures in the warmest (bio5) and minimum temperatures of the coldest months (bio6) may negatively affect it (Supplementary Fig. S4). The former can be explained by the positive correlation between the mean temperature and the effective temperature needed to complete the life cycle of the insect. The negative response to higher temperatures in the coldest months is suspected to be associated with the facultative diapause of the insect in the local climate which is crucial in terms of the survival of the overwintering adults due to the lack of suitable prey in the cold periods^16^. This suggestion supports the conclusions of studies that attributed the unsuccessful naturalization of *P*. *bioculatus* to the asynchronous seasonal cycle of predator and prey in Europe^15^. On the other hand, high temperatures attributed to climate change in the warmest and coldest months may induce phenological shifts that impede the completion of the nymphal development and successful formation of diapause before the disadvantageous periods of the year^30–32^. The results of the ENM correspond to the conditions characteristic to the climatic zones where the species is currently distributed, thus the species will most likely invade other parts of the continent with bioclimatic environments similar to its current distribution.

**Fig. 5:**
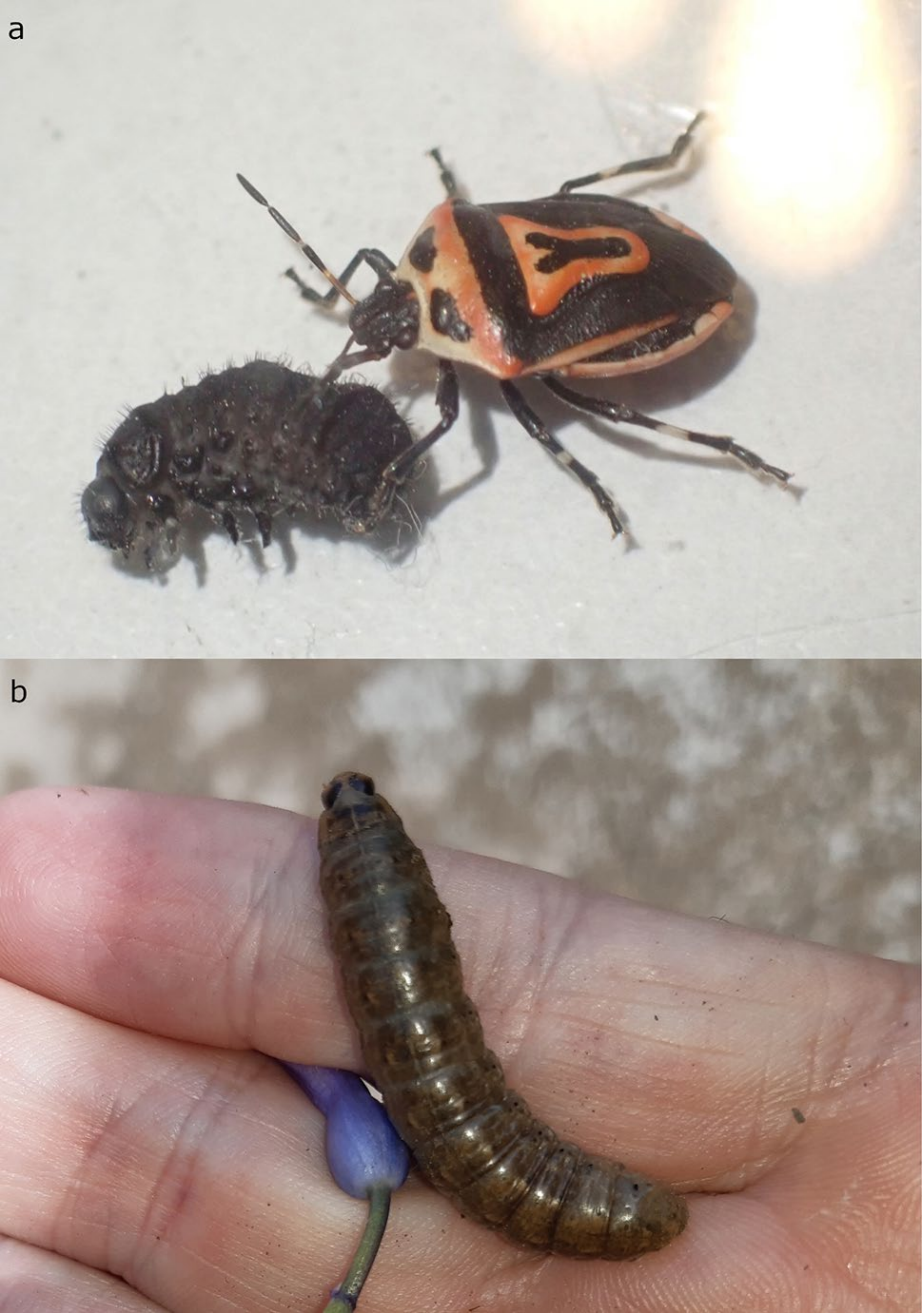
New prey records of the two-spotted stink bug in Hungary: (**a**). adult two-spotted stink bug preying on the larva of black-punctured leaf beetle (*Galeruca tanaceti*) (photo and observation by Balázs Károlyi, Budapest, Hungary), (**b**). caterpillar of turnip moth (Agrotis segetum) (associated record: PERBIO_HUN_001) (Identifier refers to the record included in Supplementary Data S1).

Most of the observations were reported from an environment with potatoes planted where the primary prey of *P. bioculatus* viz. the Colorado potato beetle (*Leptinotarsa decemlineata*) is to be found (Fig. 2). In fifteen instances predation was observed including a new prey record of the caterpillar of the noctuid moth, *Agrotis segetum* (Fig. 5a), a widely distributed lepidopteran species that is considered as agricultural pest. Furthermore, an external record was shared with the senior author of a captive adult individual feeding on the larvae of the chrysomelid beetle, *Galeruca tanaceti* (Fig. 5b). Though *P. bioculatus* was known as an oligophagous predator for decades, novel data suggest that the successful naturalization of the species in Europe is associated with a dietary drift, i.e., the utilization of alternative preys at least in adult stage (Supplementary Table S5). Laboratory experiments concluded that feeding the nymphs with alternative prey may cause higher malformation and death rates in the course of development^25^, thus *L*. *decemlineata* is the most suitable prey for the nymphal stages of *P*. *bioculatus*. However, according to the prey records it is suggested that adults effectively utilise alternative food sources, i.e., larvae of other chrysomelid beetle and caterpillars of noctuid moth species, in the absence of the Colorado potato beetle^5,24, 25^. The rapid expansion and dietary drift raise the question of how the establishment and expansion of *P*. *bioculatus* will impact European insect faunas with an emphasis on the competitive pressure put on native asopine true bugs and the prospective prey species of nature conservation importance. The topicality of the question can be explained by the case of *Harmonia axyridis*. This Asian ladybeetle species was introduced to Europe as an effective biocontrol agent of aphids, but after its naturalisation and expansion became a strong competitor and intraguild predator of native coccinellids causing serious ecological problems^33,34^. The first aspect was investigated by reviewing the habitat utilization and prey preference data available for European Asopiane (Supplementary Table S6). The compiled literature data suggests that *P*. *bioculatus* can be the competitor of most if not all native predatory stink bugs because the European fauna predominantly consists of generalist species both in terms of habitat and prey preferences. However, it must be noted that the prerequisite of this scenario is the infiltration of natural and semi-natural habitats where these species are predominantly found. The citizen science data suggests that the species is currently found in anthropogenic habitats, i.e. gardens and plantations. This can be explained by the presence of the primary prey species in these habitats and in part by the more intensive presence of contributors in such environments. However, the increasing population densities and literature data (e.g.,ref^15,22^) suggest that two-spotted stink will likely invade natural and semi-natural habitats too. The second aspect resulted in the invasion of non-anthropogenic habitats inhabited by various species allied to known prey, i.e., chrysomelid beetles and noctuid moths. These groups are of nature conservation importance at least regionally because of the limited distribution or host plant specificity of a relatively high proportion of the included species. Notable examples are the case of Serbian and Romanian noctuid moths where 35.67 and 53,84% of the species meet at least the “Near Threatened” criteria of the IUCN Red List^35^. Furthermore, a study on the Italian Chrysomelidae fauna revealed that 14.82% of the species are endemic to the Apennine Peninsula^36^. In these countries *P*. *bioculatus* is either established or suspected to settle in the future, thus their insect faunas are potentially affected by the ongoing expansion. However, it must be stressed that this thread is merely speculative serving as a call for attention and future monitoring data may prove or refute these suggestions.

Our results reveal the established and widely distributed status of the predatory stink bug, *P*. *bioculatus* in Hungary and Eastern Europe and formulate hypotheses on an ongoing westward range expansion that presumably affects regions with moderately warm climates (Fig. 4). It must be noted that the species is apparently in the expansion phase of the invasion based on the available data and, thus not to be considered in equilibrium with the environment of the invaded area^37^. Models constructed for a species in a non-equilibrium state are less accurate than those in equilibrium because of the possibility of niche shifts during the expansion^38^. In the case of the two-spotted stink bug, it is assumed that the species has found a „home away from home” in Europe, i.e., both its realized and invaded niches are part of the fundamental niche as it was found in the cases of invasive species introduced from North America to Europe and vice versa^39^. This assumption is supported by the results of previous field experiments on rearing^4,5, 15, 16^ along with similarities in the climate of the native and invaded ranges and the results of our ENM models [Supplementary data S3, S4]. However, the possibility of further shifts in the invaded range cannot be excluded, thus we want to stress that our study is an initial assessment of the recent European invasion of the two-spotted stink bug and serves as an early warning that can be clarified or even revised based on further distribution data originating from other parts of Europe. We also corroborate the hypotheses that the successful naturalization and expansion of the species on the continent is promoted by climate change and dietary drift simultaneously as suggested by previous studies from other European countries^15,21, 23^. The results of the campaign launched after the first records of the two-spotted stink bug in Hungary and the data acquired from community data-sharing platforms support the account that citizen science is a powerful tool that provides invaluable data regarding the expansions and life history characteristics of invasive species^27–29, 34^, especially in early stages of invasion.

## Methods

### Citizen science data acquisition, curation and interpretation

After the first records of *P*. *bioculatus* in Hungary (5. 10. 2023, Cserkút; 7. 10. 2023, Biharugra), we launched a citizen science campaign on the Facebook social media platform on 31.03.2024. The contributors were asked to report sightings of the species including photo documentation and description as detailed as possible of the circumstances of record viz. observations regarding habitat and behaviour. Until the last update (20.06.2024) 30 valid observations were submitted, 29 from various locations in Hungary and one from Senta, Serbia (Supplementary Data S1). Due to the characteristic appearance of *P*. *bioculatus,* no misidentified observations resulted, thus the three records with no photographic proof were included in the analysis. Submitted data was recorded using Microsoft Excel (.xlsx) assigning a unique identifier (PERBIO_HUN_001, PERBIO_HUN_002, etc.) to individual observations. Fields of the dataset consist of species name, individuals observed, date of observation, country, location, coordinates of location, habitat, observed behaviour (predation or copulation) and reference to submitted media (photos and videos).

To interpret resulted data the following categories were generated based on the content of the reports:

- Abundance: single (1 observed individual), multiple (2–10 observed individuals), mass occurrences (> 10 observed individuals)
- Habitat: garden (with potato planted), potato plantation, indoor
- Behaviour: n.a. (no specific behaviour was observed, e.g., the insect was crawling on the substrate or was stationary), mating, predation, both [multiple individuals were observed either mating, feeding or both simultaneously (e.g., Fig. 2b)].

Results were visualized with Scimago Graphica software.

### Distribution data processing and ENM

European distribution database of *P*. *bioculatus* – a sum of 145 records – was obtained from published literature, records on citizen science platforms (iNaturalist, izeltlabuak.hu), and records submitted via e-mail from citizen science contributors to the call published on Facebook. A summarized list of records (including data sources) can be found in Supplementary Table S2. Localities of previously attempted introductions were not included because the establishment cannot be proven. 26 records from literature and citizen science observations where locality is unequivocally identifiable at least to the municipality level were georeferenced postfactum using Google Earth software. Records were grouped into three time periods (pre-2010, 2010–2020, and post-2020) to visualize and interpret range expansion.

For ecological niche modelling a combined dataset of 798 geographically unique records(^26,40^, Supplementary Table S2) including both the native and invaded range of the species following the conclusions of Broennimann & Guisan^41^. 145 records originated from the invaded range (Europe) as detailed above and 653 records represented the native area (North America) of the species. Eight environmental variables, annual mean temperature (bio1), mean diurnal range (bio2), the maximum temperature of the warmest month (bio5), minimum temperature of the coldest month (bio6), annual precipitation (bio12), precipitation of the wettest (bio13) and driest (bio14) months, and elevation above sea level (bio_elev) were obtained from the WorldClim 2.0 raster dataset of 30’ (~ 1 km) spatial resolution^42^ to predict distribution of *P*. *bioculatus*. The variables were chosen based on the results of previous studies which concluded that these environmental dimensions are the most relevant in the prediction of the distribution of various invasive arthropods^43–45^ and were used with good results in the case of predaceous true bugs^46^. Ecological niche modelling was performed with the Maxent software (version 3.4.3, November 2020)^47^. Modelling was conducted with 10 replications (replicated run type: bootstrap), and random test percentages were set to 25. All models were performed with 10000 maximum background points, regularisation multiplier 1, 1000 maximum iteration, 10−^5^ convergence threshold and logistic output format following the suggestions of the above-cited articles. The accuracy and performance of the models were evaluated by using threshold-independent receiver operation characteristic (ROC)^48^ analysis where the area under the curve (AUC) takes up values between 0 and 1, and models with AUC value higher than 0.75 are accepted as robust models^49^.

Distribution data and results of ENM were interpreted and maps were generated with QGIS 3.34.7 “Prizren” geographic information system using WorldClim altitude raster layer^39^ and Koeppen–Geiger 1-km climatic classification maps raster layers^50^ to project data.

## Supplementary Information

Supplementary Table1.

Supplementary Information.

Supplementary Figure1.

Supplementary Figure2.

Supplementary Table2.

Supplementary Table3.

## Data Availability

The datasets used and analysed during the current study are included in this published article [and its supplementary information files], associated media (i.e., photographic proof of records) is available from the corresponding author on reasonable request.

## References

[1] DeClercq, P. Predaceous stinkbugs (Pentatomidae: Asopinae). In *Heteroptera of economic importance* (eds Schaefer, C. W. & Panizzi, A. R.) (Cambridge University Press, 2000).

[2] Péricart, J. *Hémiptères Pentatomoidea Euro-Méditerranéens* (Fédération Française des Sociétés de Sciences Naturelles, 2010).

[3] EFSA Panel On Plant Health (PLH) *et al.* Pest categorisation of *Leptinotarsa decemlineata*. *EFSA J.* **18**(12), e06359 (2020). 33354235 10.2903/j.efsa.2020.6359 PMC7748030

[4] Jasič, I. On the life cycle of *Perillus bioculatus*. *Acta Entomol. Bohemoslov* **72**(6), 383–390 (1975).

[5] Kil’, V. I., Ismailov, V. Y., Agas’eva, I. S., Besedina, E. N. & Fedorenko, E. V. Biological characteristics of the predatory two-spotted stink bug *Perillus bioculatus* F. and PCR study of its phylogeny. *Russ. Agric. Sci.* **38**(3), 218–221. 10.3103/s106836741203010x (2012). 10.3103/s106836741203010x

[6] Weissbecker, B., Van Loon, J. J., Posthumus, M. A., Bouwmeester, H. J. & Dicke, M. Identification of volatile potato sesquiterpenoids and their olfactory detection by the two-spotted stinkbug *Perillus bioculatus*. *J. Chem. Ecol.* **26**, 1433–1445. 10.1023/a:1005535708866 (2000). 10.1023/a:1005535708866

[7] Franz, J. Beobachtungen über die natürliche Sterblichkeit des Kartoffelkäfers *Leptinotarsa decemlineata* (Say) in Canada. *Entomophaga* **2**, 197–212. 10.1007/bf02372437 (1957). 10.1007/bf02372437

[8] Franz, J. & Szmidt, A. Beobachtungen beim züchten von Perillus bioculatus (Fabr.) (Heteropt., Pentatomidae), einem aus Nordamerika importierten räuber des kartoffelkäfers. *Entomophaga* **5**, 87–110. 10.1007/bf02374399 (1960). 10.1007/bf02374399

[9] Jermy, T. The introduction of *Perillus bioculatus* into Europe to control the colorado beetle. *EPPO Bulletin* **10**, 475–479. 10.1111/j.1365-2338.1980.tb01733.x (1980). 10.1111/j.1365-2338.1980.tb01733.x

[10] Trouvelot, B. Recherches sur les parasites et predateurs attaquant le doryphore en Amerique du Nord. *Ann. Epiphyt.* **17**, 408–445 (1932).

[11] Le Berre, J. R. & Portier, G. Utilisation d’un hétéroptère Pentatomidae *Perillus bioculatus* (Fabr.) dans la lutte contre le doryphore *Leptinotarsa decemlineata* (Say): Premiers résultats obtenus en France. *Entomophaga* **8**, 183–190. 10.1007/bf02376087 (1963). 10.1007/bf02376087

[12] Tremblay, E. & Zouliamis, N. Concluding data on the introduction, biology and use of Perilloides bioculatus in southern Italy. In *Studies of the working party of the CNR for the integrated control of animal pests of plants*: XXVII. (1968)

[13] Lakocy, A. Observations on the resistance to DDT of the blossom beetle (*Meligethes aeneus*) and the Colorado potato beetle (*Leptinotarsa decemlineata*) in Poland. *J. Plant Prot. Res.* **9**(1), 157–170 (1967).

[14] Bjegović, P. The natural enemies of the Colorado potato beetle (*Leptinotars decemlineata* Say) and attempts on its biological control in Yugoslavia. *Zaštita Bilja* **21**, 97–111 (1971).

[15] Ismailov, V. Y. & Agas’eva, I. S. Predatory Bug *Perillus bioculatus* Fabr, A new view at possibilities of acclimatization and prospects of use. *Zashch. Karan. Rast* **2**, 30–31 (2010).

[16] Saulich, A. K. & Musolin, D. L. Seasonal Cycles of Pentatomoidea 1. In *Invasive stink bugs and related species (Pentatomoidea): Biology, higher systematics, semiochemistry, and management* (ed. McPherson, J. E.) (CRC Press, 2018).

[17] Mikloš, I. Contribution la connaissance de faune entomologique predateur sur les peupliers dans la R.S. de Croatie. *Šumarski list* **5–6**, 224–231 (1967).

[18] Kivan, M. Some observations on *Perillus bioculatus* (F.) (Heteroptera: Pentatomidae) a new record for the entomofauna of Turkey. *Turkish J. Entomol.* **28**(2), 95–98 (2004).

[19] Simov, N., Langourov, M., Grozeva, S. & Gradinarov, D. New and interesting records of alien and native true bugs (Hemiptera: Heteroptera) from Bulgaria. *Acta Zool. Bulg.* **64**(3), 241–252 (2012).

[20] Radac, I. A. & Teodorescu, M. First records of *Mustha spinosula* and *Perillus bioculatus* (Heteroptera: Pentatomidae) Romania. *Trav. Du Mus. Natl. Hist. Nat. Grigore Antipa*10.3897/travaux.64.e64664 (2021). 10.3897/travaux.64.e64664

[21] Derjanschi, V. & Elisovetskaya, D. 2013 Predatory shield bug Perillus bioculatus F. (Hemiptera, Pentatomidae) in the Republic of Moldova: acclimatization or natural colonization? In *Actual problems of protection and sustainable use of the animal world diversity*, 124–125

[22] Protić, L., Čkrkić, M. M., Stojanović, A. & Smiljanić, D. Distribution of *Perillus bioculatus* (Fabricius, 1775) in Serbia. *Bull. Nat. Hist. Mus.* **15**, 121–136 (2022).

[23] Nadaždin, B. Šeat New data on *Perillus bioculatus* (Heteroptera: Pentatomidae) in Serbia: Do climate change and a new food source contribute to the true bug expansion?. *Acta Entomol. Serbica* **27**(2), 83–90. 10.5281/zenodo.7225252 (2022). 10.5281/zenodo.7225252

[24] Tarla, S. & Tarla, G. Detection of *Perillus bioculatus* (F.) (Heteroplera: Pentatomidae) on a new host in Anatolia. *Canad. Entomol.* **127**, 195–212 (2018).

[25] Elisovetcaia, D., Derjanschi, V., Agas’eva, I. & Nefedova, M. Some results of breeding a predatory stink bug of *Perillus bioculatus* F (Hemiptera, Pentatomidae) in the Republic of Moldova. *BIO web of conferences, EDP Sciences* **21**, 24 (2020). 10.1051/bioconf/20202100024

[26] GBIF.org *GBIF Occurrence Download.*10.15468/dl.wsec36 (2024).

[27] Maistrello, L., Dioli, P., Bariselli, M., Mazzoli, G. L. & Giacalone-Forini, I. Citizen science and early detection of invasive species: Phenology of first occurrences of Halyomorpha halys in Southern Europe. *Biol. Invasions* **18**(11), 3109–3116. 10.1007/s10530-016-1217-z (2016). 10.1007/s10530-016-1217-z

[28] Páll-Gergely, B. *et al.* Realtime social networking service rapidly reveals distributions of non-indigenous land snails in a European capital. *Bioinvasions Rec.* **8**(4), 782–792. 10.3391/bir.2019.8.4.06 (2019). 10.3391/bir.2019.8.4.06

[29] Turóci, Á., Fehér, Z., Krízsik, V. & Páll-Gergely, B. Two new alien slugs, Krynickillus melanocephalus Kaleniczenko, 1851 and *Tandonia kusceri* (H. Wagner, 1931), are already widespread in Hungary. *Acta Zool. Acad. Sci. Hung.* **66**(3), 265–282. 10.1710/azh.66.3.265.2020 (2020). 10.1710/azh.66.3.265.2020

[30] Bale, J. S. & Hayward, S. A. L. Insect overwintering in a changing climate. *J. Exp. Biol.* **213**(6), 980–994. 10.1242/jeb.037911 (2010). 20190123 10.1242/jeb.037911

[31] Harvey, J. A., Heinen, R., Gols, R. & Thakur, M. P. Climate change-mediated temperature extremes and insects: From outbreaks to breakdowns. *Glob. Change Biol.* **26**(12), 6685–6701. 10.1111/gcb.15377 (2020). 10.1111/gcb.15377 PMC7756417 33006246

[32] Buckley, L. B. Temperature-sensitive development shapes insect phenological responses to climate change. *Curr. Opin. Insect Sci.* **52**, 100897. 10.1016/j.cois.2022.100897 (2022). 35257968 10.1016/j.cois.2022.100897

[33] Roy, H. E. *et al.* The harlequin ladybird, Harmonia axyridis: Global perspectives on invasion history and ecology. *Biol. invasions* **18**, 997–1044 (2016). 10.1007/s10530-016-1077-6

[34] Werenkraut, V., Baudino, F. & Roy, H. E. Citizen science reveals the distribution of the invasive harlequin ladybird (Harmonia axyridis Pallas) in Argentina. *Biol. Invasions* **22**, 2915–2921. 10.1007/s10530-020-02312-7 (2020). 10.1007/s10530-020-02312-7

[35] Stojanović, D. V., Ćurčic, S. B., Ćurčic, B. P. M. & Makarov, S. E. The application of IUCN Red List criteria to assess the conservation status of moths at the regional level: A case of provisional Red List of Noctuidae (Lepidoptera) in Serbia. *J. Insect Conserv.* **17**, 451–464. 10.1007/s10841-012-9527-7 (2013). 10.1007/s10841-012-9527-7

[36] Biondi, M., Urbani, F. & D’Alessandro, P. Endemism patterns in the Italian leaf beetle fauna (Coleoptera, Chrysomelidae). *Zookeys* **332**, 177–205. 10.3897/zookeys.332.5339 (2013). 10.3897/zookeys.332.5339 PMC3805321 24163584

[37] Sakai, A. K. *et al.* The population biology of invasive species. *Annu. Rev. Ecol. Syst.* **32**(1), 305–332. 10.1146/annurev.ecolsys.32.081501.114037 (2001). 10.1146/annurev.ecolsys.32.081501.114037

[38] Václavík, T. & Meentemeyer, R. K. Equilibrium or not? Modelling potential distribution of invasive species in different stages of invasion. *Divers. Distrib.* **18**(1), 73–83. 10.1111/j.1472-4642.2011.00854.x (2011). 10.1111/j.1472-4642.2011.00854.x

[39] Aravind, N. A. *et al.* Niche shift in invasive species: is it a case of “home away from home” or finding a “new home”?. *Biodivers. Conserv.* **31**, 2625–2638. 10.1007/s10531-022-02447-0 (2022). 10.1007/s10531-022-02447-0

[40] GBIF.org *GBIF Occurrence Download*. 10.15468/dl.2jak55 (2024).

[41] Broennimann, O. & Guisan, A. Predicting current and future biological invasions: Both native and invaded ranges matter. *Biol. Lett.* **4**(5), 585–589. 10.1098/rsbl.2008.0254 (2008). 18664415 10.1098/rsbl.2008.0254 PMC2610080

[42] Fick, S. E. & Hijmans, R. J. (2017) WorldClim 2: New 1-km spatial resolution climate surfaces for global land areas. *Int. J. Climatol.* **37**(12), 4302–4315. 10.1002/joc.5086 (2017). 10.1002/joc.5086

[43] De Meyer, M. *et al.* Ecological niche and potential geographic distribution of the invasive fruit fly Bactrocera invaders (Diptera, Tephritidae). *Bull. Entom. Res. London* **100**(1), 35–48 (2010). 10.1017/S0007485309006713 19323851

[44] Kumar, S., Graham, J., West, A. M. & Evangelista, P. H. Using district-level occurrences in MaxEnt for predicting the invasion potential of an exotic insect pest in India. *Comput. Electron. Agric.* **103**, 55–62. 10.1016/j.compag.2014.02.007 (2014). 10.1016/j.compag.2014.02.007

[45] Hill, M. P., Gallardo, B. & Terblanche, J. S. A global assessment of climatic niche shifts and human influence in insect invasions. *Glob. Ecol. Biogeogr.* **26**(6), 679–689. 10.1111/geb.12578 (2017). 10.1111/geb.12578

[46] Solhjouy-Fard, S. & Sarafrazi, A. Patterns of niche overlapping and richness among Geocoris species (Hemiptera: Geocoridae) in Iran. *Biocontrol Sci. Techn.* **26**(9), 1197–1211. 10.1080/09583157.2016.1182619 (2016). 10.1080/09583157.2016.1182619

[47] Phillips, S. J., Anderson, R. P., Dudík, M., Schapire, R. E. & Blair, M. E. Opening the black box: An open-source release of maxent. *Ecography* **40**(7), 887–893. 10.1111/ecog.03049 (2017). 10.1111/ecog.03049

[48] Elith, J. *et al.* Novel methods improve prediction of species’ distributions from occurrence data. *Ecography* **29**(2), 129–151. 10.1111/j.2006.0906-7590.04596.x (2006). 10.1111/j.2006.0906-7590.04596.x

[49] Pearce, J. & Ferrier, S. Evaluating the predictive performance of habitat models developed using logistic regression. *Ecol. Model.* **133**(3), 225–245. 10.1016/s0304-3800(00)00322-7 (2000). 10.1016/s0304-3800(00)00322-7

[50] Beck, H. E. *et al.* High-resolution (1 km) Köppen-Geiger maps for 1901–2099 based on constrained CMIP6 projections. *Sci. Data* **10**(1), 724. 10.1038/s41597-023-02549-6 (2023). 37872197 10.1038/s41597-023-02549-6 PMC10593765

