# Supplementary figures and images for: Past, present and future of the two-spotted stink bug (Perillus bioculatus) in Europe revealed by citizen science
Source: Sci Rep. 2024 Sep 14;14:21494. doi: 10.1038/s41598-024-72501-0 (PMC11401935; doi:10.1038/s41598-024-72501-0)

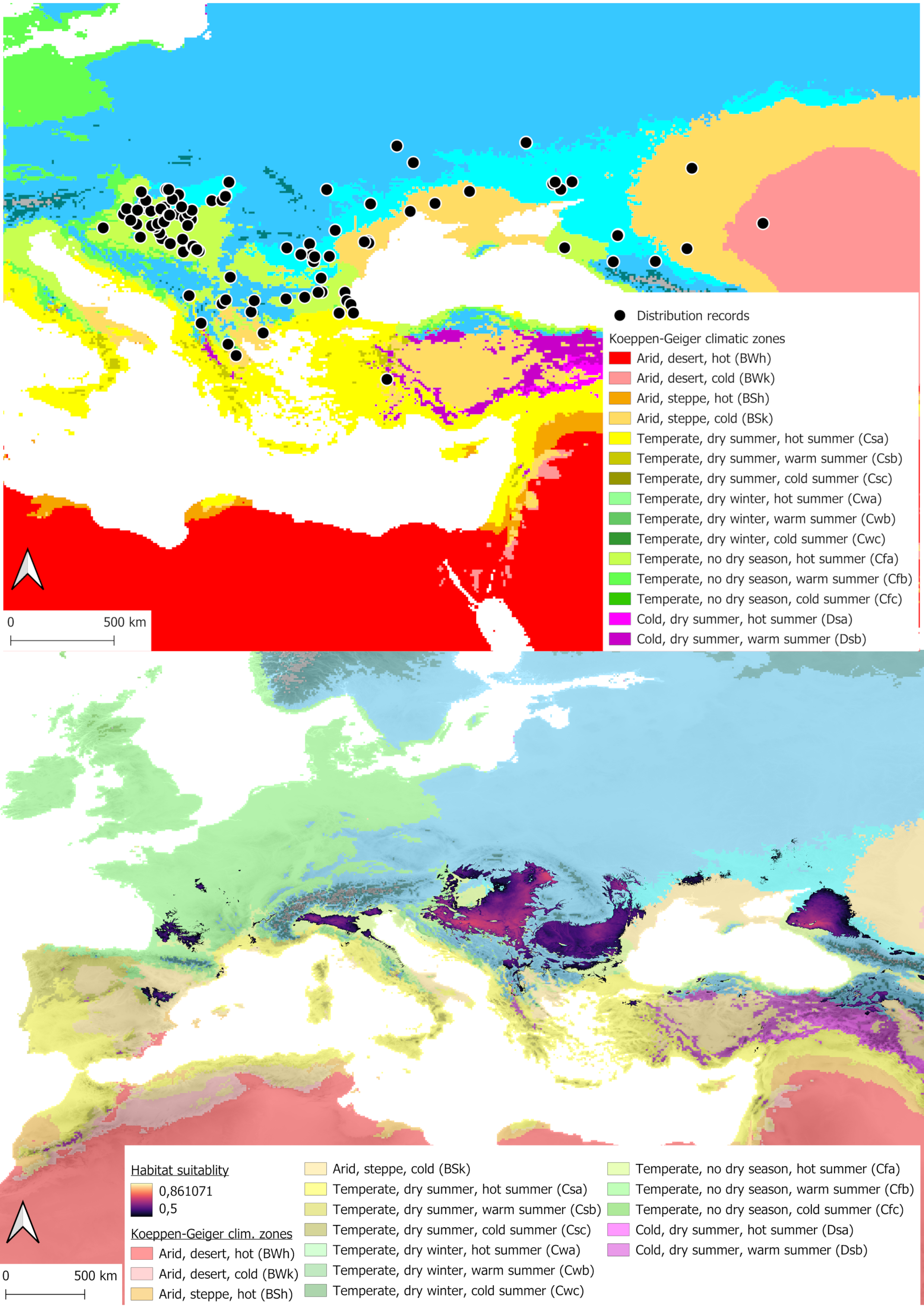

Supplement: Supplementary file 3 — Supplementary Figure1. [file 41598_2024_72501_MOESM3_ESM.tif]

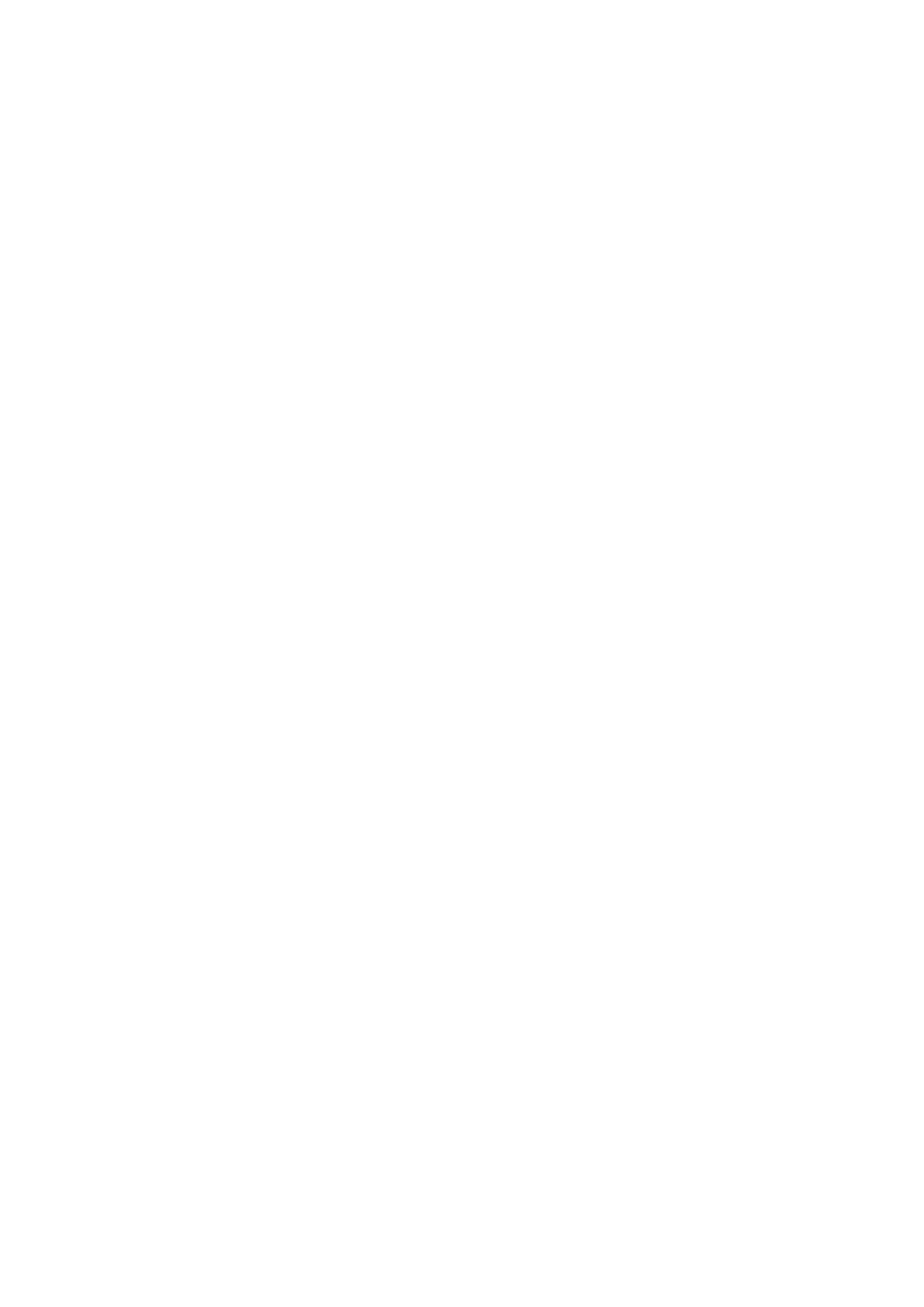

Supplement: Supplementary file 4 — Supplementary Figure2. [file 41598_2024_72501_MOESM4_ESM.tif]
